# Latent gene network expression underlies partial re-evolution of a polyphenic trait in the worker caste of ants

**DOI:** 10.7554/eLife.110148

**Published:** 2026-08-18

**Authors:** Angelly Vasquez-Correa, Johanna Arnet, Travis Chen, Ehab Abouheif

**Affiliations:** 1 Center for Evolutionary and Organismal Biology, Women’s Hospital, School of Medicine, Zhejiang University Hangzhou China; 2 McGill University, Department of Biology Montreal Canada; University at Buffalo, State University of New York United States; New York University United States

**Keywords:** eye-antenna disc, ocelli, ants, reversion, polyphenism, latent developmental potential, Other

## Abstract

Polyphenisms–where alternative phenotypes develop from a single genome in response to environmental cues–are not only widespread in nature, but also occur at multiple levels of biological organization, from cells to individuals to societies. Polyphenism is thought to promote phenotypic diversification through the gain, loss, and re-evolution of alternative phenotypes. After the origin of a polyphenism, one of the alternative phenotypes often retains the developmental capacity to produce the ancestral trait, thereby permitting the other to evolve rapidly. Yet, little is known about the developmental processes underlying the re-evolution of polyphenic traits, and how they may produce phenotypic diversification. Here, we address this question by focusing on the caste polyphenism in ant societies, which produces a winged queen caste and a wingless worker caste in a single colony in response to environmental cues. We show, in a hyperdiverse group of ants, that a caste-specific trait called the ocelli (three simple eyes on the dorsal head) is always present across queen castes but was lost and partially re-evolved multiple times, giving rise to novel patterns (one ocelli) in the worker castes. Surprisingly, we discovered that a hidden (latent) expression of the ocelli gene regulatory network in worker castes that lost ocelli underlies the partial re-evolution of ocelli in this group. We therefore propose that latent developmental potentials may generally persist across polyphenic systems, including ant castes, and may facilitate the partial re-evolution of novel phenotypic patterns.

## eLife digest

The re-emergence of traits lost over evolutionary time has long fascinated biologists and sparked debate. Yet we still do not fully understand the developmental and evolutionary mechanisms that allow lost traits to reappear. This question is particularly relevant in species that exhibit polyphenism – the ability of the same genome to produce different forms in response to environmental signals.

Ants are a striking example of polyphenism in action. Within a single colony, the same genome can produce morphologically distinct queens, males and workers. One of the most remarkable polyphenic traits is the presence of ocelli, three simple light-sensing eyes on the top of the head. Queens and males consistently possess ocelli, but in workers they have been repeatedly lost and gained during evolution. Across different species, workers may have one, two or all three ocelli, suggesting that the trait has evolved independently multiple times.

This remarkable evolutionary flexibility led Vasquez-Correa et al. to propose that the developmental programme for forming ocelli remains dormant in workers, making it easier for the trait to reappear. To test this idea, the researchers reconstructed the evolutionary history of worker ocelli across the hyperdiverse ant subfamily Formicinae.

Their ancestral state reconstruction revealed a single evolutionary gain of worker ocelli, followed by multiple losses and independent reappearances. To understand how this repeated re-evolution occurred, the researchers examined ant head development, focusing on the eye-antennal disc. This tissue gives rise to the eyes, antennae, ocelli and head capsule.

Using hybridization chain reaction, a technique that visualizes gene activity in fixed tissues, they tracked the expression of genes involved in ocellus development throughout development. Remarkably, even in workers that lacked ocelli, key developmental genes were briefly active, suggesting that the underlying gene regulatory network remained intact. This retained developmental programme appears to have enabled the repeated evolution of ocelli in Formicinae workers and soldiers.

Polyphenism is widespread in nature, occurring at many levels of biological organization, from individual cells to complex societies. The findings of Vasquez-Correa et al. suggest that dormant developmental programmes may commonly persist after traits are lost, providing hidden evolutionary potential. If similar latent gene activity exists in other organisms, it could help explain how lost traits repeatedly re-evolve in new forms, offering fresh insights into the origins of evolutionary novelty.

## Introduction

Polyphenism is a form of developmental plasticity where alternative phenotypes develop from a single genome in response to environmental cues (Nijhout, 2003). It is a phylogenetically widespread feature of plants and animals that has evolved at different levels of biological organization (Hanna et al., 2024; West-Eberhard, 1989; West-Eberhard, 2003). For example, at the population level, the mouth form polyphenism in nematode worms produces alternative big tooth (omnivorous) or small tooth (bactivorous) mouth forms that develop in response to pheromones, crowding, salt concentration, temperature, and culturing substrate (Bento et al., 2010; Bose et al., 2012; Ragsdale et al., 2013; Werner et al., 2018). At the colony-level, caste polyphenism in eusocial insects produces morphologically differentiated queen and worker castes that develop in response to temperature and nutrition (Chandra et al., 2018; Evans and Wheeler, 2001; Korb, 2025; Rajakumar et al., 2024). And finally, at the cellular-level, polyphenism occurs within a multicellular individual, where a single genome gives rise to differentiated cell-types during development, such as between germline and somatic cells, in response to internal cues like morphogen gradients (Brunet and King, 2017; Davison and Michod, 2021; Devlin et al., 2023). Polyphenism has been proposed to promote, at the macroevolutionary scale, phenotypic diversification through the gain, loss, and re-evolution of alternative phenotypes (West-Eberhard, 2003). This is based on the idea that, once a polyphenic trait originates, one of the alternative morphs retains the capacity to produce the trait in the genome while the other is freer to evolve. This hypothesis has received support from a comparative study of mouth form polyphenism across 90 species of nematode worms showing a phylogenetic association between the gain and loss of alternative mouth form phenotypes and the phenotypic diversification of mouth parts (Susoy et al., 2015). Another supporting example is fat synthesis in parasitic wasps, which revealed an association between developmental plasticity and the loss and subsequent re-evolution of fat synthesis in one species (Peters et al., 2017; Visser et al., 2010; Visser et al., 2021). Yet, the underlying developmental and genetic processes facilitating the gain, loss, and re-evolution of polyphenic traits remain poorly understood. (Forni et al., 2026; Sommer, 2020; West-Eberhard, 2003).

Here, we address this question by focusing on caste polyphenism in the eusocial colonies of ants, which consists of a morphological division of labor between a winged reproductive queen caste and wingless non-reproductive worker caste in almost all 16,962 valid described ant species (AntWeb, 2026; Ward, 2014). The differential expression of polyphenic traits, such as wings, that develop in queens but not workers, are called ‘caste-specific’ traits (Miura, 2005). It has been shown that polyphenic traits, including caste polyphenism in ants, are produced during development by the differential expression of highly conserved gene regulatory networks (GRN) in response to environmental cues (Abouheif and Wray, 2002; Béhague et al., 2018; Casasa et al., 2021; Davidson et al., 2023; Lenuzzi et al., 2023; Rajakumar et al., 2024; Vizueta et al., 2025). However, how the expression of these GRNs influences the evolution of caste-specific traits in ants remains unknown.

**Figure 1.**
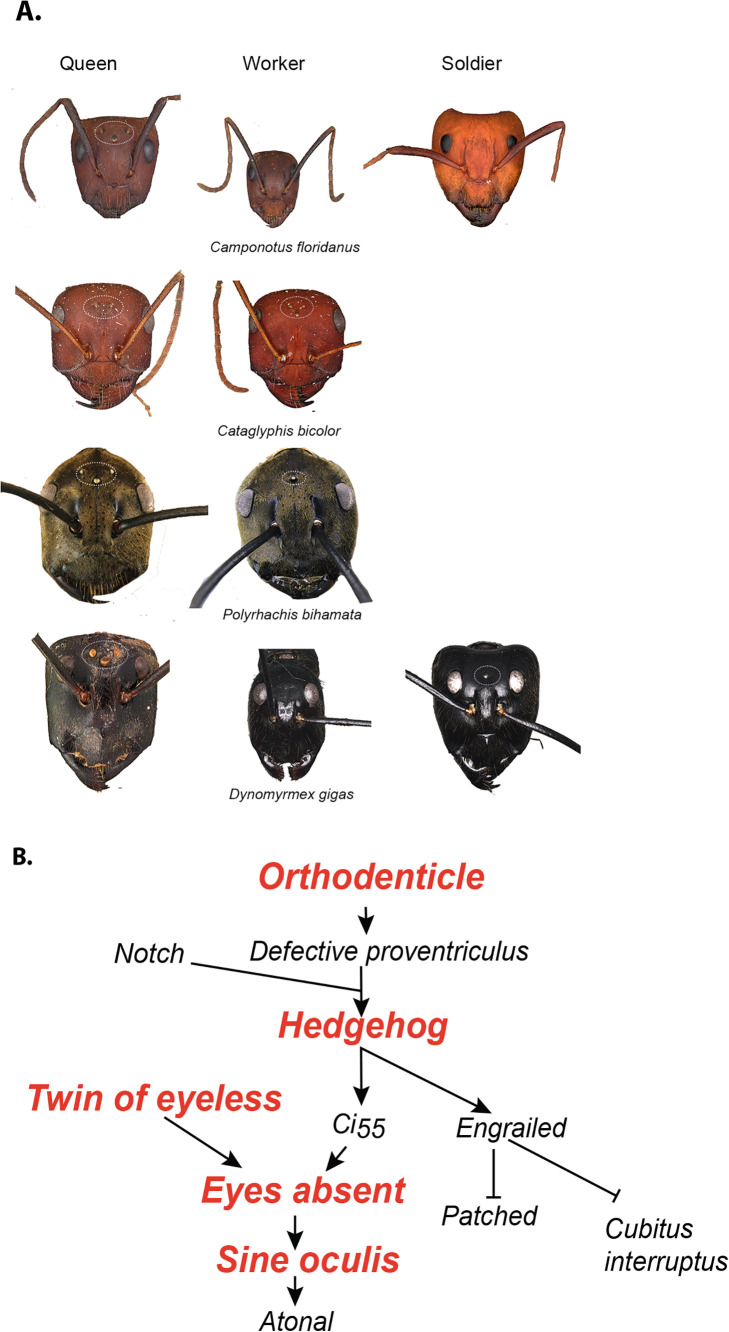
The presence and absence of ocelli in queens and workers across ants. (**A**) Ocelli develop in the winged reproductive castes across species of Formicinae ants exemplified by *C. floridanus*, *C. bicolor, P. bihamata,* and *D. gigas* (white dashed circles). Ocelli in the worker caste of formicine ants (white dashed circles) are evolutionarily labile, showing no ocelli (*C. floridanus*), all three ocelli in all individuals of the worker caste (*C. bicolor*), or only one ocellus in only the soldiers (*P. bihamata* and *D. gigas*). (**B**) Schematic representation of the ocelli GRN in *Drosophila melanogaster* adapted from Figure 5A from Jean-Guillaume and Kumar, 2022. The genes investigated in this study are highlighted in orange. Arrowheads indicate activation, and bars indicate repression. Queens are scaled to 1 mm, and workers and soldiers are scaled according to the queen of each species. Asterisks indicate that *C. floridanus* was used for gene expression studies. Photos from Antweb (AntWeb, 2026).

Here we focus on the ocelli, which are three small single-lens eyes on the dorsal head of most flying insects. Ocelli complement the function of the compound eyes by mediating orientation using polarized light and in the synchronization of daily activity (Buschbeck and Bok, 2023; Krapp, 2009). We investigate the evolution of ocelli in a hyperdiverse subfamily of ants (Formicinae), where they are universally present in the winged reproductive caste (queens and males) as three large ocelli that aid in mating flights and dispersal (Moser et al., 2004; Narendra et al., 2016). In contrast, ocelli in the wingless worker caste are evolutionarily labile, showing dramatic variation across species in the presence/absence or number of ocelli in the worker caste (Johnson and Rutowski, 2022; Narendra et al., 2016; Narendra and Ribi, 2017; Schwarz et al., 2011; Figure 1A). In some species, adult workers completely lack ocelli, such as in *Camponotus floridanus*, while in others they are present and vary in number–there are species whose adult workers have all three ocelli or just one single ocellus, and these can be present in all or in only a subset of workers (Figure 1A). For example, workers in *Cataglyphis bicolor* have all three ocelli, which function in light sensing and navigation, acting as a celestial compass that provides crucial directional information (Fent and Wehner, 1985; Figure 1A). In contrast, all workers of *Polyrachis bihamata* have just a single medial ocellus (Hung, 1967; Figure 1A), and in workers of *Dinomyrmex gigas*, a single medial ocellus evolved only in a subset of individuals in the worker caste called ‘soldiers’ (or major workers) with large heads, but are absent in other individuals called ‘minor workers’ with small heads (AntWeb, 2026; Figure 1A). How this dramatic variation in ocelli in the worker caste of formicine ants has evolved remains poorly understood.

This evolutionary lability of ocelli across the worker castes of formicine ants also provides an opportunity to understand how the GRN underlying development of ocelli influenced the evolution of this caste-specific trait. Ants are holometabolous insects, in which adult body parts develop from imaginal discs, semi-independent clusters of cells in the larvae (Held, 2002; Koch and Abouheif, 2019). In the fruit fly *D. melanogaster*, ocelli develop from the eye-antenna imaginal disc located at the ventral region of the head capsule. The eye-antenna imaginal disc also gives rise to the head capsule, eye, antenna, and maxillary palps (Held, 2002). *D. melanogaster* is the only insect where the GRN underlying ocelli development has been well characterized at the third larval stage (Blanco et al., 2009; Domínguez-Cejudo and Casares, 2015; Sabat et al., 2017; Figure 1B). The gene *orthodenticle* (*otd*; formerly known as *ocelli-less*) is a selector gene that is necessary for specifying the ocellar region in the developing head capsule. *otd* expression in the ocellar domain, together with other genes like *hedgehog* (*hh*), initiates the development of the ocellar region and the three ocelli (two lateral ocelli and one medial ocellus) in the eye-antennal disc. The activation of these genes regulates the expression of downstream genes, such as *otd* regulating the expression of *defective proventriculus* (*dve*; Blanco et al., 2009; Jean-Guillaume and Kumar, 2022; Yorimitsu et al., 2011), whereas *hedgehog* (*hh*) activates a portion of the retinal determination network, such as *eyes absent* (*eya*), *twin of eyeless* (*toy*), *sine oculis* (*so),* and *atonal* (*ato*). These retinal determination genes have been shown to be regulated by independent regulatory enhancers from the compound eye (Blanco et al., 2009; Blanco et al., 2010; Jean-Guillaume and Kumar, 2022; Figure 1B). Because the eye-antennae disc and the ocelli GRN have only been well characterized in *D. melanogaster*, it remains unknown whether they are conserved in ants.

To understand how the GRN underlying ocelli development may have influenced the evolution of this caste-specific trait, we first inferred the evolutionary history of ocelli in adult workers across the Formicinae using ancestral state reconstruction. We then characterized the eye-antennae disc in ants using three genes, *eyeless* (*ey*), *distal-less* (*dll*), and *otd-1*, which are known to mark the eyes (*ey*), the antenna (*dll*), and the head capsule and ocelli (*otd-1*). This characterization allowed us to investigate the expression of five key genes in the ocelli GRN, *otd-1, hh, toy, eya,* and *so*, during development of the winged and wingless castes across two formicine species.

## Results

### Partial reversion to a single ocellus occurs three times independently within the tribe Camponotini (Formicinae)

**Figure 2.**
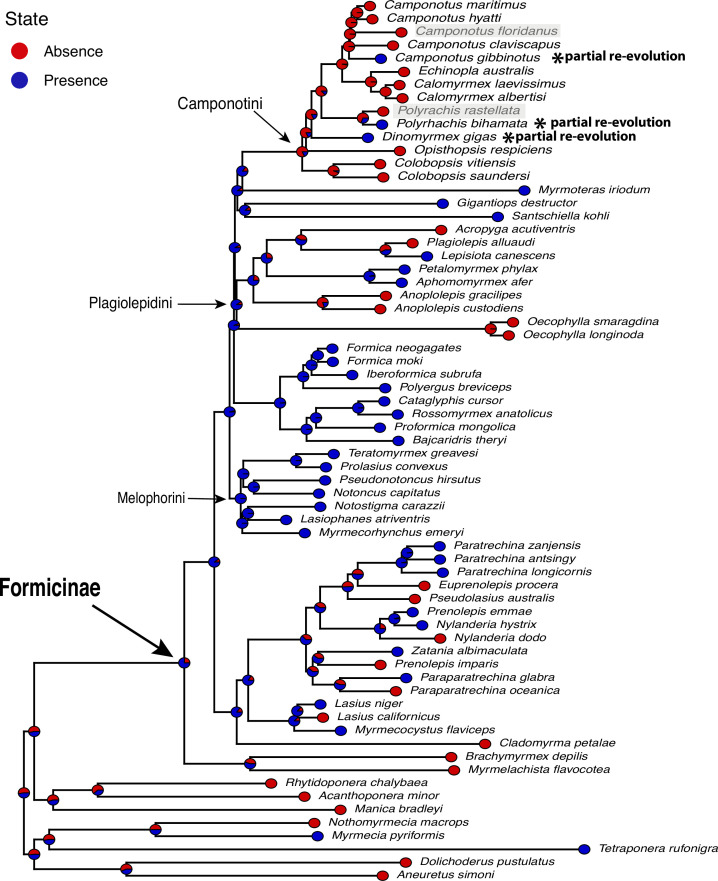
Ancestral state reconstruction of ocelli reveals three well-supported reversions of ocelli in the worker caste in the tribe Camponotini. Maximum clade credibility tree of formicine ants from Blaimer et al., 2015. Ancestral state reconstruction for the presence (blue-colored circles) and absence (red-colored circles) of ocelli based on stochastic character mapping. Each pie chart for the nodes represents the posterior probabilities, scaled by the weight of evidence for each model. Species used to analyze ocelli GRN expression are highlighted in gray, and the species that re-evolved one ocellus is indicated as partial re-evolution. Three tribes within the Formicinae are marked (Melophorini, Plagiolepidini, Lasiini, Myrmelachistini and Camponotini) by arrows.

We first performed an ancestral state reconstruction to infer the evolutionary history of worker ocelli across the subfamily Formicinae. Our ancestral state reconstruction inferred a single re-gain of worker ocelli at the base or early within the Formicinae (Figure 2). Subsequent to the gain of ocelli early in the evolution of the Formicinae, our analysis inferred a single, well-supported loss of worker ocelli at the base of the tribe Camponotini (Figure 2). Following this single loss, we inferred three (well-supported) independent and partial re-evolution to a single medial ocellus in three different genera: *Camponotus gibbinotus*, *P. bihamata*, and *D. gigas* (Figure 2). In *P. bihamata*, the single ocellus occurs in all workers in the colony, whereas in *C. gibbinotus* and *D. gigas* the single ocellus occurs only in the large-headed soldiers (Figure 1). We therefore investigated the developmental role of the ocelli GRN underlying these independent partial reversions to a single medial ocellus in this tribe of ants.

### A fate map characterizing the development of the head capsule, antennae, eyes, and ocelli within the eye-antenna disc in ants

**Figure 3.**
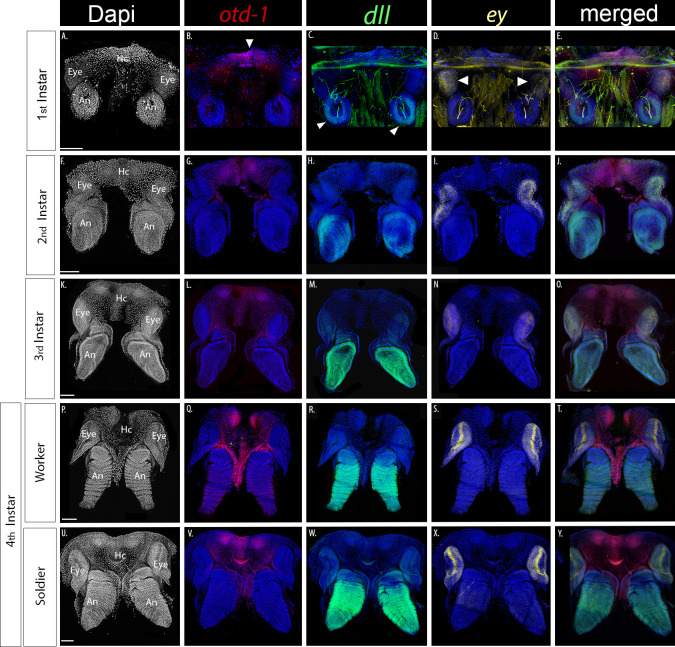
Characterizing development of the eye-antenna imaginal disc in worker castes of *C. floridanus* using *orthodenticle-1 (otd-1), distal-less (dll*), and *eyeless (ey*) gene expression to mark the developing head capsule and ocelli, antenna, and eyes. Fluorescent images in panels **A, F, K, P, U** represent the development of the entire eye-antenna imaginal disc marked with the nuclear stain DAPI across all four larval stages, where the head capsule region is labeled as ‘Hc’, the antennal region is labeled as ‘An,’ and the eye region is labeled as ‘Eye’. Panels **B, G, L, Q**, and **V** represent the development of the head capsule (Hc) marked by the genes *orthodenticle-1 (otd-1* in magenta); panels **C, H, M, R**, and **W** represent the antennal region marked by *distal-less* (*dll*) in green color; and panels **D, I, N, S**, and **X** represent the eyes (Eye) is *eyeless (ey*) (yellow); (**A–E**) First instar, images are to scale (white bar in **A**). Note: the green or yellow staining outside of the structures highlighted by the white arrows in panels **C, D**, and **E** is background noise. (**F–J**) Second instar, images are to scale (white bar in **F**), (**K–O**) third instar, images are to scale (white bar in **K**), (**P–T**) fourth instar worker-destined larvae, images are to scale (white bar in **P**). (**U–Y**) Fourth instar soldier-destined larvae, images are to scale (white bar in **U**). Scale bars = 100 µm.

To understand whether the GRN underlying the development of ocelli influenced the reversions of this caste-specific trait in workers, we first had to characterize the development of the eye-antenna disc in ants. In *D. melanogaster*, the eye, antennae, maxillary palps, ocelli, and head capsule develop from the eye-antenna disc, which is segregated into regions marked by the expression of highly conserved developmental genes (Haynie and Bryant, 1986; Held, 2002). In the Florida carpenter ant *C. floridanus*, we found that, similar to *D. melanogaster*, expression of *otd-1* marks the precursor regions of the head capsule and ocelli (Figure 3—figure supplement 1), *ey* marks the precursor regions of the eyes, and *dll* marks the precursor regions of the antennae (Figure 3). During the first larval instar, expression of *otd-1* emerges primarily in the head capsule in the middle part of the disc between the antenna and compound eye (Figure 3B). In contrast, *dll* and *eya* expression delineate the precursor regions of the antenna and eye (Figure 3C and D). During the second and third larval instar, *otd-1* is expressed in the developing head capsule and ocelli in the medial region of the disc (Figure 3G and L), while *dll* is confined to the antenna and *ey* to the compound eye region (Figure 3H, I, M and N). Finally, during the fourth (final) larval instar, a developmental threshold mediated by juvenile hormone acts as a switch point to determine whether larvae will develop either into a minor worker or soldier (MacMillan et al., 2025). Once larvae have been determined, expression patterns of *otd-1*, *dll,* and *ey* in worker-destined larvae (Figure 3P–T) or soldier-destined larvae (Figure 3U–Y) remain expressed in the same regions as in the second and third instars (Figure 3Q–T, V–Y). Together, our characterization shows that the eye-antennae disc and the regional identities within it, including the precursor region of the head capsule, ocelli, eyes, and antennae, are conserved in ants relative to *Drosophila*.

**Figure 3—figure supplement 1.**
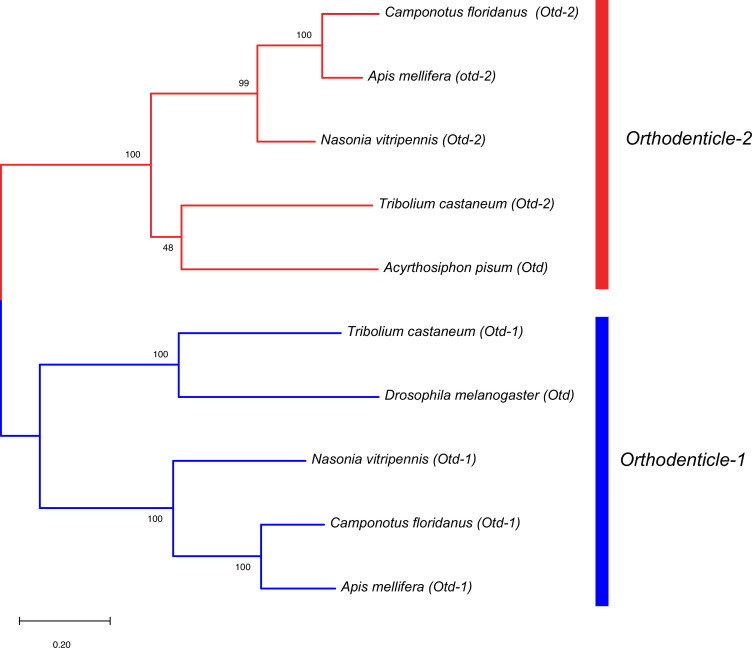
Simplified Gene tree based on maximum likelihood showing relationships between *orthodenticle (otd)* orthologs in *D. melanogaster*, *Apis mellifera*, *Nasonia vitripennis*, *Tribolium castaneum, Acythosiphon pisum, and C. floridanus*. Branch values are bootstrap support (%). Colors: *otd-2* (red), *otd-1* (blue).

### Expression of the ocelli GRN is conserved in winged reproductive castes but is latent in species whose adult workers completely lack ocelli

**Figure 4.**
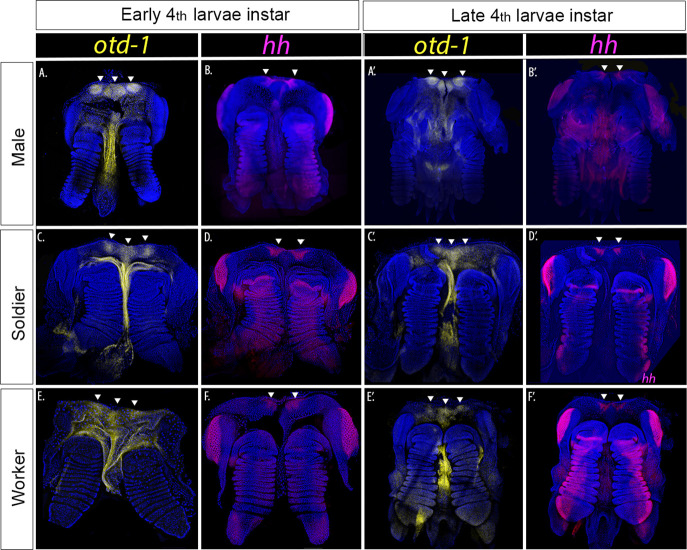
Latent expression of *otd-1* and *hh* genes in the ocelli GRN in workers and soldiers of *C. floridanus* during the 4th larval instars. Expression of *orthodenticle-1 (otd-1*) is yellow, and *hedgehog (hh*) is magenta. Early 4th instar; (**A, B**) males (**C, D**) soldiers and (**E, F**) workers. Late 4th instar: (**A,’ B’**) males (**C’, D’**) soldiers and (**E’, F’**) workers. Images are not to scale.

**Figure 5.**
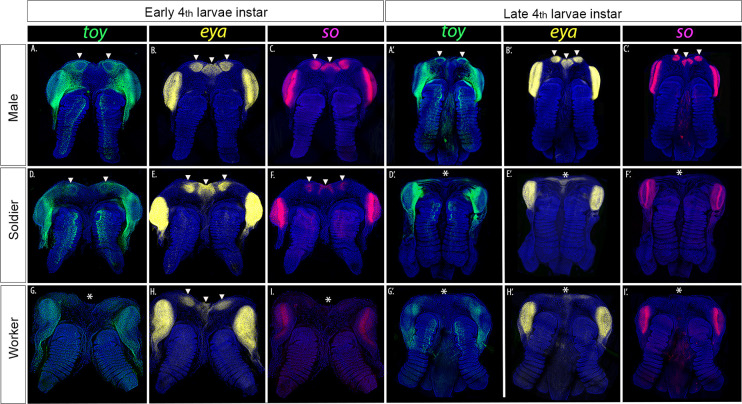
Latent expression of *toy*, *eya*, and *so* within the ocelli GRN in the developing workers and soldiers of *C.* *floridanus*. Expression of *toy* (green), *eya* (yellow)*,* and *so* (magenta). Early 4th instar; (**A–C**) males, (**D–F**) soldiers, and (**G–I**) workers. Late 4th larvae stage; (**A’–C’**) males, (**D’–F’**), soldiers, and (**G’–I’**). Images are not to scale.

We next asked whether the ocelli GRN is conserved in the winged reproductive caste (males) relative to *Drosophila* and whether it is expressed in workers that entirely lack ocelli as adults. We address these questions using two species *C. floridanus* and *Polyrachis rastellata*. We chose these two species because *C. floridanus* is closely related to *C. gibbinotus* and *P. rastellata* is closely related to *P. bihamata*, which are two of the species that our ancestral state reconstruction inferred independent partial reversions to single medial ocellus in the worker caste (Figure 2). In the winged male caste of *C. floridanus*, we found that *otd-1, eya,* and *so* are expressed where the three ocelli will develop, while *toy* and *hh* are expressed in the inter-ocellar region (the tissue that separates the three ocelli) (see white arrowheads in Figure 4A–B’ and Figure 5A–C’). Because these genes are similarly expressed within the ocellar region within the eye-antennal disc of *D. melanogaster*, we infer that the ocelli GRN is conserved in the winged reproductive castes in ants.

**Figure 6.**
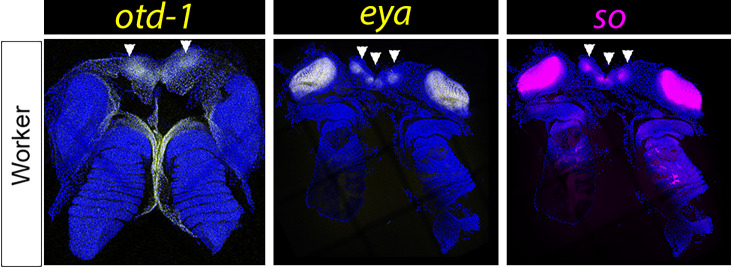
Latent expression of the ocelli GRN in the eye-antennal disc in worker larvae of *Polyrachis rastellata* at 4th larval stages. Expression of selected genes *otd-1* (yellow), *eya* (yellow), and *so* (magenta) at early 4th instar larvae. Images are not to scale.

Surprisingly, we discovered that the ocelli GRN remains latently expressed in minor worker- and soldier-destined larvae of *C. floridanus*, which completely lack ocelli as adults. In soldier-destined larvae, all five genes remain expressed in the ocellar region within the eye-antennal disc at the beginning of the last larval instar (Figures 4C, D, 5C–F). By the end of this instar, *otd-1* and *hh* remain expressed (Figure 4C’ and D’), but *toy*, *eya*, and *so* are either down-regulated or absent relative to their expression in the compound eye region (Figure 5D’–F’). In minor worker-destined larvae, three of the five genes (*otd-1*, *hh, eya*) remain expressed in the ocellar region during the early part of the last larval instar (Figures 4E, F, 5H), whereas *toy* and *so* are expressed in the compound eye region but absent (interrupted) in the ocellar region (Figure 5G and I). Furthermore, in *P. rastellata*, whose worker caste is composed of similarly sized individuals with no subcastes, we found that *otd-1*, *eya*, and *so* remain expressed in the ocellar region within the eye-antennal disc during the early part of the last larval instar (Figure 6). Together, our results show that despite the absence of ocelli in adult workers for millions of years, the expression of the ocelli GRN remains latent during larval development.

**Figure 7.**
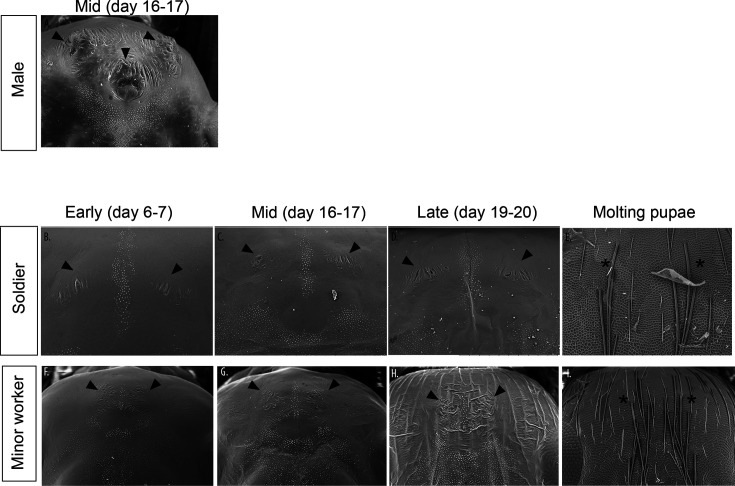
Development of rudimentary ocelli in worker and soldier pupae of *C. floridanus*. (**A**) SEM showing ocelli development in males at mid-stages of pupal development. SEM showing development of rudimentary ocelli on (**B–E**) soldiers and (**F–I**) minor workers during early (day 6–7), mid (day 16–17), and late (19-20) pupal development. These ocelli rudiments disappear prior to adult stage (**E–I**).

Finally, we performed Scanning Electron Microscopy (SEM) in *C. floridanus* male, soldier, and minor worker pupae. In minor worker and soldier pupae, we discovered the existence of rudimentary ocelli that appear at the beginning of pupal development, continue to be elaborated, and then are eliminated before they molt into adult workers (Figure 7). These ocelli rudiments are highly reduced relative to the fully functional ocelli found in male pupae. Finally, the pattern and timing of development of ocelli rudiments in the minor worker and soldier pupae coincide with the spatial expression and timing of interruption of the latent expression of the ocelli GRN. In soldier-destined larvae, ocelli GRN expression is interrupted later in development than in minor worker-destined larvae, and consequently, the ocelli rudiments in soldier pupae continue to develop longer and are more elaborated relative to those in minor worker pupae (Figure 7B–D, F–H). Therefore, expression of the latent ocelli GRN in the eye-antennae disc results in the development of ocelli rudiments in worker pupae and are then eliminated in adult workers.

## Discussion

Our developmental and evolutionary data provide evidence that the latent expression of genes in workers lacking ocelli as adults is part of a latently expressed ocelli GRN, which likely facilitated at least three independent evolutionary reversions of this trait in the worker caste of species within the Camponotini clade. The latent expression patterns of genes in the ocelli region of developing workers lacking ocelli as adults are the same as in males that will develop fully functional ocelli but are only interrupted late in larval development. Furthermore, the timing and pattern of these late interruptions coincide with the degree of development of rudimentary ocelli in minor worker and soldier pupae before they disappear in adults (Figure 7). This indicates that, although the expression of these genes is latent, they still retain the capacity to produce rudimentary ocelli in the pupal stage before they disappear in adults. And finally, although *eya*, *toy*, and *so*, are part of both the ocelli and compound eye GRNs in *Drosophila*, the ocelli GRN has its own distinct identity and the genes within this GRN have distinct regulatory elements and are selectively regulated (Jean-Guillaume and Kumar, 2022; Zimmerman et al., 2000). In ants, our data show that the selector gene for compound eye development in insects (*eyeless* / *Pax-6*) is expressed in the compound eyes and not ocelli, and the selector gene for ocelli (*otd-1*) is expressed in the ocelli and not compound eyes. We further show that the latent expression of *toy*, *eye*, and *so* are downregulated or absent in the ocellar region, but at the same time, are strongly. expressed in the regions of the compound eyes. Therefore, the mutually exclusive expression of the selector genes *eyeless* / *Pax-6* in the compound eyes and *otd-1* in ocelli suggests that, like in *Drosophila*, the compound eye and ocelli GRNs have distinct identities, ultimately leading to differential expression of downstream genes and production of different cell types; compound eyes are produced from multiple imaging-forming facets, while ocelli are produced from a single lens (Buschbeck and Bok, 2023; Jean-Guillaume and Kumar, 2022; Mishra et al., 2021). Altogether, our data show that the expression of these genes in workers lacking ocelli as adults is part of a latently expressed ocelli GRN.

Several hypotheses may explain how the ocelli GRN came to be latently expressed and maintained in developing workers that lack ocelli as adults. Perhaps the most simplistic hypothesis proposes that the presence of functional ocelli in adult queen and male castes maintains the ocelli GRN intact in the genome by keeping it under positive natural selection. This hypothesis assumes that this, as a side consequence, leads to expression of the ocelli GRN in the worker castes lacking ocelli. However, caste determination between queens and workers occurs through the action of a developmental threshold or switch, where a continuous environmental cue is translated into discrete phenotypic outcomes (Abouheif, 2021; MacMillan et al., 2025; Qiu et al., 2022; Rajakumar et al., 2024; Schultner et al., 2023). Once caste determination has occurred, the genome is expressed differentially during the developmental trajectories of queens and workers (Abouheif, 2021; Abouheif and Wray, 2002; Barkdull and Moreau, 2023; Béhague et al., 2018; Chandra et al., 2018; Khila and Abouheif, 2010; Qiu et al., 2022; Vizueta et al., 2025). These trajectories are decoupled, and consequently, can evolve largely independently (Abouheif, 2021; Powell et al., 2020; Vong et al., 2025). The dramatic variation in the number, size, and presence/absence of ocelli in worker castes across the Formicinae (see Figure 1) supports the largely independent evolution of ocelli in workers from those in queens, which always develop three ocelli. Furthermore, we observe similar patterns of variation in the wings and ovaries between queen and worker castes across ant species (Cronin et al., 2013; Khila and Abouheif, 2010; Monnin and Peeters, 2008; Rajakumar et al., 2018; Rajakumar et al., 2012). Therefore, while the presence of ocelli in males and queens maintains the ocelli GRN in the genome and creates a potential for expression of this GRN in developing workers lacking ocelli, this cannot solely explain how this latent expression became actualized (released and fixed) and what maintained it over millions of years.

One hypothesis for how this ocelli GRN became latently expressed and has been maintained in workers lacking ocelli is pleiotropy, which potentially results from the multiple roles that genes within the ocelli GRN play within the same imaginal disc (the eye-antennal disc). For instance, *otd-1* and *hh* also determine the regional identity of the head capsule, while *hh*, *toy*, *eya,* and *so* also play key roles in compound eye development in specifying structures such as optic lobes, cone differentiation, and rhabdomeres development (Blanco et al., 2009; Domínguez-Cejudo and Casares, 2015; Jean-Guillaume and Kumar, 2022). Another example is the highly conserved developmental regulatory gene *sonic hedgehog* (*shh*), which plays a key role in limb development across animals. The vestigialization of hindlimbs in snakes and therefore the re-evolution of hindlimbs in extinct species across the phylogeny of the group is thought to be maintained during development through pleiotropic enhancers that drive *shh* expression. This means that the same enhancer (ZRS) that drives *shh* expression in the external genitalia also drives it (pleiotropically) in the developing limb region of snakes (Leal and Cohn, 2018). Future studies should attempt to explore whether expression of these conserved genes in multiple regions of the eye-antennal disc is driven by shared enhancers. Alternatively, we cannot rule out the hypothesis that the ocelli GRN has been co-opted to play a novel, yet currently unknown, function during worker larval development. Recent discoveries on the evolution of the wing GRN in ants provide support for this hypothesis. The wings, another nearly universal caste-specific trait in ants, develop in the reproductive male and queen caste, but are halted in the worker caste in response to environmental cues (Abouheif and Wray, 2002). The wing GRN is also found to be latently expressed in the wingless worker caste of ants and was thought to be functionless (Abouheif and Wray, 2002). However, it was recently discovered that the latent expression of this wing GRN in wingless worker caste acquired a novel function to generate big-headed soldiers in the hyperdiverse ant genus *Pheidole* (Rajakumar et al., 2018; Rajakumar et al., 2012).

Finally, our inference that this latent expression of the ocelli GRN in workers facilitated the partial reversion to a single medial ocellus is supported by: (1) the close phylogenetic relationship between species that lack ocelli in adult workers but retain a latent expression of the ocelli GRN (*C. floridanus* and *P. rastellata*), and those that underwent a partial phylogenetic reversion to a single medial ocellus (*C. gibbinotus*, *D. gigas,* and *P. bihamata*); (2) the presence of a developmental capacity or potential of the latent ocelli GRN expression to produce rudimentary ocelli in the pupal stage of *C. floridanus* workers that completely lack ocelli as adults; (3) the ability to experimentally induce only one, only two, or all three ocelli in similarly sized adult workers that normally lack them by applying high doses of Juvenile Hormone (JH) to worker-destined larvae in the ant *Monomorium pharaonis* (Li et al., 2024); and finally (4) in nature, the rare induction of a single medial ocellus by mermithid parasites in soldiers of *Pheidole pallidula* that typically lack ocelli in natural colonies (Laciny et al., 2019; Passera, 1976). The natural or experimental induction of worker individuals with only a single medial ocellus in different ant species also supports the inference that the single medial ocellus can be developmentally dissociated from the other two lateral ocelli. This suggests that reversion can facilitate the appearance of novel patterns of ocelli development in the workers if selected for.

In *C. floridanus* and *P. rastellata*, there is a latent ocelli GRN expression for all 3 ocelli, providing a springboard to facilitate the partial phylogenetic reversion to a single medial ocellus in *C. gibbinotus*, *P. bihamata,* and *D. gigas*. In the genus *Polyrhachis*, however, some species in the same subgenus as *P. bihamata*, such as *P. bellicosa*, have three ocelli. Because the phylogenetic relationships within this subgenus have yet to be resolved, the independent reversion of ocelli in the ancestor of this subgenus may have resulted either in a single ocellus as reflected in *P. bihamata* or in three ocelli as reflected in *P. bellicosa,* and two ocelli were subsequently lost giving rise to the single medial ocellus in *P. bihamata* (Hung, 1967). These possibilities further reinforce the different evolutionary pathways by which this latent potential may facilitate novelty after reversion.

Future functional, genomic, and comparative analyses of the ocelli GRN between larval stages, individuals within the worker caste, and species will ultimately reveal the architecture of the ocelli GRN and whether its underlying enhancers and promoters are modular or pleiotropic. This, in combination with manipulations of insect hormones, such as JH and ecdysone, would also elucidate whether variation in the size, presence/absence, number of ocelli is regulated by continuous or switch-like developmental mechanisms. And finally, determination of the organismal and ecological function of the ocelli will be important to understand the adaptive significance of the latent expression of the ocelli GRN at both the individual and colony-level.

More broadly, our findings suggest that the ancestral and latent GRN expression (also known as ancestral developmental potential) we observed may generally underlie polyphenic systems, including caste-specific traits in ants and other eusocial organisms. We therefore propose that ancestral developmental potentials facilitate the re-evolution of polyphenic traits (West-Eberhard, 2003), and when these potentials facilitate only the partial re-evolution of alternative phenotypes, novel phenotypic patterns appear. We hope our findings not only inspire future work testing these proposals in polyphenic organisms, but also in non-polyphenic ones, where the polyphenism occurs at the cellular level but not at the level of the whole organism. Here, the cellular polyphenism produces alternative cell-types from a single genome in response to internal cues, such as morphogen gradients within the organism. If alternative cell types retain homologs or serial homologs of specific traits (Jackman et al., 2025; Lynch, 2023), then this raises the possibility that ancestral and latent developmental potentials may generally facilitate re-evolution of alternative cell types in multicellular organisms.

## Materials and methods

**Key resources table:** 

Reagent type (species) or resource	Designation	Source or reference	Identifiers	Additional information
Gene (Camponotus floridanus)	eyeless distal-less hedgehog eye absent sine oculis twin of eyeless orthodenticle	GenBank	XM_025414466 XM_025412727.1 XM_011262474.3 XM_011262474.3 XM_011252868.3 XM_011268499.3 XM_020028684.2	
Commercial assay or kit	Hybridization Chain Reaction in situ hybridization probes	Molecular Instruments		
Software, algorithm	R software Fiji Photoshop Illustrator Tracer Geneious MEGA 12 alpa			

### Ant maintenance and collection

Colonies of *C. floridanus* were collected at Gainesville (Florida, USA), and *P. rastellata* were collected at Mae Tang (Chiang Mai, Thailand), under the following import permit numbers from the Canadian Food Inspection Agency: Florida (P-2016–02921, P-2018–00809) and Thailand (Ants from Asia, P-2019–00011). Ants were maintained in plastic boxes with glass test tubes filled with water-constrained cotton wool. They were fed mealworms and the Bhatkar–Whitcomb diet (Bhatkar and Whitcomb, 1970). Colonies were maintained at 25 °C with 60% humidity in complete darkness. All colonies were housed in growth chambers at McGill University’s Phytotron Facility under Plant Pest Containment Level 1 certification numbers PC-2016–057 and PC-2018–265 from the Canadian Food Inspection Agency. None of the species collected are endangered as determined by their absence on the IUCN Red List of Threatened Species (https://www.iucnredlist.org/, and search Formicidae) or were collected on protected lands.

### Larvae fixation and *in-situ* HCR

Gene sequences were obtained from NCBI GenBank database (Sayers et al., 2022) using genome BLAST against the assembled *C. floridanus* genome: *eyeless (ey*; XM_025414466), *distal-less (dll*; XM_025412727.1), *hedgehog (hh*; XM_011262474.3), *eye absent (eya*; XM_025414466), *sine oculis (so;* XM_011252868.3), and *twin of eyeless (toy*; XM_011268499.3) genes. For *otd*, two paralogs of the gene were found in ants (XM_020028684.2 and XM_025415314.1), which is a result of a gene duplication event that has also been reported in wasps, bees, and beetles (Lynch et al., 2006; The Honeybee Genome Sequencing Consortium, 2006; Zattara et al., 2017). The two *otd* paralogs sequences were then aligned by multiple sequence alignment using all of the known *orthodenticle* related sequences in insects: *D. melanogaster* (NM_001369965.1), *A. mellifera: otd-1* (XM_026446161.1), *otd-2* (XM_006571236.3), *Nasonia vitripennis: otd-1* (XM_008212114.4), *otd-2* (XM_031926951.2), and *T. castaneum otd-1* (XM_008192467.2), *otd-2* (XM_008192470.2), and *A. pisum* (XP_008180802.1). To determine the *otd-1* paralog to *D. melanogaster* (*otd-1*), a maximum likelihood gene tree was inferred using genetic distance model Hasegawa Kishino Yano (HKY) and 500 bootstrap replicates as incorporated in MEGA12 alpha (Kumar et al., 2024). Probes corresponding to all genes were chosen for the hybridization chain reaction experiments using the fluorescence Hairpins (B1 546, B2 488, B3 647) synthesized by Molecular Instruments.

First, second, third, and fourth larval instars of soldier and minor worker-destined larvae and fourth instar of male destined larvae of *C. floridanus* and worker larvae of *P. rastellata* were collected and subsequently fixed in a PEM 4% formaldehyde solution for 2 hr at room temperature. Fixed samples were then dehydrated progressively in methanol baths (25%, 50%, 75% methanol for 15 min each, and 100% overnight at 4 °C) and stored in 100% methanol at −30 °C until use. All gene expression analyses were conducted by in situ Hybridization Chain Reaction (HCR), following the protocol for HCR (v3.0 protocol; Schwarzkopf et al., 2021). After the tissue was pre-hybridized in a prewarmed Probe Hybridization Buffer (Molecular Instruments) for 30 min at 37 °C and incubated with HCR probes in a Probe Hybridization Buffer overnight at 37 °C. Tissues were washed the next day in a prewarmed Probe Wash Buffer four times, 15 min each, and washed in 5 X SSCT (UltraPure 20XSSC Buffer, Invitrogen, diluted in water) three times for 5 min at room temperature. Tissues were pre-amplified in Amplification Buffer (Molecular Instruments) for 30 min at room temperature and incubated with snap-cooled HCR hairpins in Amplification Buffer overnight at room temperature. Tissues were then washed with 5 X SSCT at room temperature twice for 5 min, for 30 min, and once for 5 min before being mounted on glycerol-DAPI 80%.

### Microscopy

Confocal imaging was used to describe gene expression using a Leica SP8 confocal microscope. Fiji (Schindelin et al., 2012) was used for image processing. Scanning electron microscopy (SEM) was done on a Hitachi TM3030 Scanning Electron Microscope.

### Evolution of ocelli in the formicine clade

The evolution of ocelli on workers across the subfamily Formicinae was inferred using ancestral reconstruction (ASE) for discrete traits incorporated in the R package Phytools 4.3.3 (Revell, 2024). The ASE analysis was based on the UCE70 phylogeny for the clade Formicinae published by Blaimer et al., 2015. The species *Camponotus floridanus* and *P. bihamata* were added manually to the phylogeny. To determine the presence or absence of ocelli in workers, photographs of the studied species from the database AntWeb, Version 8.114 were used (AntWeb, 2026). The observations from the database were contrasted with published information from the literature (Appendix 1—table 2). Ocelli were classified as present in the worker caste if individuals exhibit any of the three ocelli (two lateral and one medial ocellus). In the case of the presence of worker polymorphism, ocelli were classified as present if any one of the three ocelli was present within any of the worker subcastes. Whereas the absence was the complete lack of ocelli across workers and soldiers.

Four separate models of ocelli evolution were tested for each character in phytools: Equal rate ‘ER’, all transitions rate different ‘ARD’, and an irreversible model allowing only transitions between presence and absence, and another irreversible model allowing only transitions between absence and presence. We compared the fit of our models by computing Akaike information criterion (AIC) and Akaike weights and conducting pairwise likelihood ratio tests. The new function incorporated in phytools 4.3.3, *simmap,* was used to generate stochastic character maps under each of the four models tested (Appendix 1—table 1). The stochastic mapping that resulted from the stochastic simulation represented the frequencies that are equal to the weight of evidence supported by each model (Revell, 2024).

## Data Availability

All data reported in this paper are provided in Appendix 1—table 2. This paper does not report original code. Any additional information required to reanalyze the data reported in this paper is available from the corresponding author upon request.

## Additional files

MDAR checklist

## Appendix 1

**Appendix 1—table 1.** Model selection under maximum likelihood estimation implemented in phytools (Revell, 2024). The results are ordered by decreasing Akaike Weights (w).

Model	log(L)	d.f.	AIC	weight
Equal Rates	–41.808109	1	85.6162173	0.70944317
Unequal Rates	–41.720603	2	87.441205	0.28485677
Irreversible: Absence to Presence	–46.813104	1	95.6262081	0.00475637
Irreversible: Presence to Absence	–48.430553	1	98.8611065	0.00094368

**Appendix 1—table 2.** Database with the references used in this study for presence and number of ocellus (1) and absence (0) of ocellus.

Subfamily	Genus	Species	Ocelli Count	References
outgroup	*Acanthoponera*	*minor*	0	AntWeb.org
Formicinae	*Acropyga*	*acutiventris*	0	AntWeb.org
outgroup	*Aneuretus*	*simoni*	0	AntWeb.org
Formicinae	*Anoplolepis*	*custodiens*	0	AntWeb.org
Formicinae	*Anoplolepis*	*gracilipes*	0	AntWeb.org
Formicinae	*Aphomomyrmex*	*afer*	3	AntWeb.org
Formicinae	*Bajcaridris*	*theryi*	3	Santschi, 1936
outgroup	*Brachymyrmex*	*depilis*	0	AntWeb.org
Formicinae	*Calomyrmex*	*albertisi*	0	AntWeb.org
Formicinae	*Calomyrmex*	*laevissimus*	0	AntWeb.org
Formicinae	*Camponotus*	*floridanus*	0	AntWeb.com
Formicinae	*Camponotus*	*gibbinotus*	1	AntWeb.org
Formicinae	*Camponotus*	*hyatti*	0	MacKay and Mackay, 2002
Formicinae	*Camponotus*	*maritimus*	0	Ward, 2005
Formicinae	*Colobopsis*	*saundersi*	0	AntWeb.org
Formicinae	*Colobopsis*	*vitiensis*	0	Mann, 1920
Formicinae	*Cataglyphis*	*cursor*	3	AntWeb.org
outgroup	*Cladomyrma*	*petalae*	0	Agosti, 1991
outgroup	*Dolichoderus*	*pustulatus*	0	AntWeb.org
Formicinae	*Dinomyrmex*	*gigas*	1	AntWeb.org
Formicinae	*Echinopla*	*australis*	0	AntWeb.org
Formicinae	*Euprenolepis*	*procera*	0	Lapolla, 2009
Formicinae	*Formica*	*moki*	3	Cole, 1943
Formicinae	*Formica*	*neogagates*	3	AntWeb.org
Formicinae	*Gigantiops*	*destructor*	3	Smith, 1858
Formicinae	*Iberoformica*	*subrufa*	3	Antwiki- genus
Formicinae	*Lasiophanes*	*atriventris*	3	AntWeb.org
Formicinae	*Lasius*	*californicus*	0	AntWeb.org
Formicinae	*Lasius*	*niger*	3	AntWeb.org
Formicinae	*Lepisiota*	*canescens*	3	Sharaf et al., 2020
outgroup	*Manica*	*bradleyi*	0	AntWeb.org
outgroup	*Myrmecia*	*pyriformis*	3	AntWeb.org
Formicinae	*Myrmecocystus*	*flaviceps*	3	AntWeb.org
Formicinae	*Myrmecorhynchus*	*emeryi*	3 (*soldiers and media, absent in minors*)	Wheeler, 1917
outgroup	*Myrmelachista*	*flavocotea*	0	AntWeb.org
Formicinae	*Myrmoteras*	*iriodum*	3	AntWeb.org
outgroup	*Nothomyrmecia*	*macrops*	0	AntWeb.org
Formicinae	*Notoncus*	*capitatus*	3	AntWeb.org
Formicinae	*Notostigma*	*carazzii*	3	AntWeb.org; Emery, 1911
Formicinae	*Nylanderia*	*dodo*	0	Lapolla et al., 2011
Formicinae	*Nylanderia*	*hystrix*	3	Kallal and LaPolla, 2012
Formicinae	*Oecophylla*	*longinoda*	0	AntWeb.org
Formicinae	*Oecophylla*	*smaragdina*	0	Cole and Jones, 1948
Formicinae	*Opisthopsis*	*respiciens*	0	AntWeb.org
Formicinae	*Paraparatrechina*	*glabra*	3	AntWeb.org
Formicinae	*Paraparatrechina*	*oceanica*	0	AntWeb.org
Formicinae	*Paratrechina*	*antsingy*	3	LaPolla and Fisher, 2014
Formicinae	*Paratrechina*	*longicornis*	3	AntWeb.org
Formicinae	*Paratrechina*	*zanjensis*	3	LaPolla et al., 2013
Formicinae	*Petalomyrmex*	*phylax*	3	Snelling, 1979
Formicinae	*Plagiolepis*	*alluaudi*	0	AntWeb.org
Formicinae	*Polyergus*	*breviceps*	3	AntWeb.org; Smith, 1947
Formicinae	*Polyrhachis*	*decumbens*	0	Kohout, 2006
Formicinae	*Prenolepis*	*emmae*	3	AntWeb.org
Formicinae	*Prenolepis*	*imparis*	0	Williams and Lapolla, 2016
Formicinae	*Proformica*	*mongolica*	3	AntWeb.org
Formicinae	*Prolasius*	*convexus*	3	McAreavey, 1957
Formicinae	*Pseudolasius*	*australis*	0	Emery, 1925
Formicinae	*Pseudonotoncus*	*hirsutus*	3	Shattuck and O’Reilly, 2013
outgroup	*Rhytidoponera*	*chalybaea*	0	AntWeb.org
Formicinae	*Rossomyrmex*	*anatolicus*	3	AntWeb.org
Formicinae	*Santschiella*	*kohli*	3	Forel, 1893
Formicinae	*Teratomyrmex*	*greavesi*	3	Shattuck and O’Reilly, 2013
outgroup	*Tetraponera*	*rufonigra*	3	Ward, 2001
Formicinae	*Zatania*	*albimaculata*	3	AntWeb.org

## References

[bib1] Abouheif E, Wray GA (2002). Evolution of the gene network underlying wing polyphenism in ants. Science.

[bib2] Abouheif E (2021). Ant caste evo-devo: it’s not all about size. Trends in Ecology & Evolution.

[bib3] Agosti D (1991). Revision of the oriental ant genus *Cladomyrma*, with an outline of the higher classification of the Formicinae (Hymenoptera: Formicidae). Systematic Entomology.

[bib4] AntWeb (2026). California Academy of Science. https://www.antweb.org.

[bib5] Barkdull M, Moreau CS (2023). Worker reproduction and caste polymorphism impact genome evolution and social genes across the ants. Genome Biology and Evolution.

[bib6] Béhague J, Fisher BL, Péronnet R, Rajakumar R, Abouheif E, Molet M (2018). Lack of interruption of the gene network underlying wing polyphenism in an early-branching ant genus. Journal of Experimental Zoology. Part B, Molecular and Developmental Evolution.

[bib7] Bento G, Ogawa A, Sommer RJ (2010). Co-option of the hormone-signalling module dafachronic acid-DAF-12 in nematode evolution. Nature.

[bib8] Bhatkar A, Whitcomb WH (1970). Artificial diet for rearing various species of ants. The Florida Entomologist.

[bib9] Blaimer BB, Brady SG, Schultz TR, Lloyd MW, Fisher BL, Ward PS (2015). Phylogenomic methods outperform traditional multi-locus approaches in resolving deep evolutionary history: a case study of formicine ants. BMC Evolutionary Biology.

[bib10] Blanco J, Seimiya M, Pauli T, Reichert H, Gehring WJ (2009). Wingless and Hedgehog signaling pathways regulate orthodenticle and eyes absent during ocelli development in *Drosophila*. Developmental Biology.

[bib11] Blanco J, Pauli T, Seimiya M, Udolph G, Gehring WJ (2010). Genetic interactions of eyes absent, twin of eyeless and orthodenticle regulate sine oculis expression during ocellar development in *Drosophila*. Developmental Biology.

[bib12] Bose N, Ogawa A, von Reuss SH, Yim JJ, Ragsdale EJ, Sommer RJ, Schroeder FC (2012). Complex small-molecule architectures regulate phenotypic plasticity in a nematode. Angewandte Chemie.

[bib13] Brunet T, King N (2017). The origin of animal multicellularity and cell differentiation. Developmental Cell.

[bib14] Buschbeck E, Bok M (2023). Distributed Vision: From Simple Sensors to Sophisticated Combination Eyes.

[bib15] Casasa S, Biddle JF, Koutsovoulos GD, Ragsdale EJ (2021). Polyphenism of a novel trait integrated rapidly evolving genes into ancestrally plastic networks. Molecular Biology and Evolution.

[bib16] Chandra V, Fetter-Pruneda I, Oxley PR, Ritger AL, McKenzie SK, Libbrecht R, Kronauer DJC (2018). Social regulation of insulin signaling and the evolution of eusociality in ants. Science.

[bib17] Cole AC (1943). A new subspecies of formica moki wheeler (hymenoptera: formicidae). American Midland Naturalist.

[bib18] Cole AC, Jones JW (1948). A study of the weaver ant, oecophylla smaragdina (fab.). American Midland Naturalist.

[bib19] Cronin AL, Molet M, Doums C, Monnin T, Peeters C (2013). Recurrent evolution of dependent colony foundation across eusocial insects. Annual Review of Entomology.

[bib20] Davidson PL, Nadolski EM, Moczek AP (2023). Gene regulatory networks underlying the development and evolution of plasticity in horned beetles. Current Opinion in Insect Science.

[bib21] Davison DR, Michod RE, Pfennig DW (2021). Phenotypic Plasticity &Amp; Evolution.

[bib22] Devlin DK, Ganley ARD, Takeuchi N (2023). A pan-metazoan view of germline-soma distinction challenges our understanding of how the metazoan germline evolves. Current Opinion in Systems Biology.

[bib23] Domínguez-Cejudo MA, Casares F (2015). Anteroposterior patterning of *Drosophila* ocelli requires an anti-repressor mechanism within the hh pathway mediated by the Six3 gene Optix. Development.

[bib24] Emery C (1911). Le genre Camponotus Mayr. Nouvel essai de sa subdivision en sous-genres. Revue Zoologique Africaine.

[bib25] Emery C (1925). Genera Insectorum.

[bib26] Evans JD, Wheeler DE (2001). Gene expression and the evolution of insect polyphenisms. BioEssays: News and Reviews in Molecular, Cellular and Developmental Biology.

[bib27] Fent K, Wehner R (1985). Oceili: a celestial compass in the desert ant cataglyphis. Science.

[bib28] Forel A (1893). Fourmis du Congo et d’autres provenances récoltées par MM. Hermann, Kohl, Luja, Mayné, etc. Revue Suisse de Zoologie.

[bib29] Forni G, Fusco G, Nicolini F, Bruce H, Luchetti A (2026). Loss, persistence and reversal of phenotypic traits. Biological Reviews of the Cambridge Philosophical Society.

[bib30] Hanna L, Boudinot BE, Liebig J, Abouheif E (2024). Programmed cell death and the origin of wing polyphenism in ants: implications for major evolutionary transitions in individuality. bioRxiv.

[bib31] Haynie JL, Bryant PJ (1986). Development of the eye-antenna imaginal disc and morphogenesis of the adult head in *Drosophila melanogaster*. The Journal of Experimental Zoology.

[bib32] Held L, Held L (2002). Imaginal Discs: The Genetic and Cellular Logic of Pattern Formation.

[bib33] Hung ACF (1967). A revision of the ant genus polyrhachis at the subgeneric level (hymenoptera: formicidae). Transactions of the American Entomological Society.

[bib34] Jackman WR, Lynch VJ, Gibert Y (2025). Challenging and redefining Dollo’s law of evolution: re-appearance of lost structures. BMC Ecology and Evolution.

[bib35] Jean-Guillaume CB, Kumar JP (2022). Development of the ocellar visual system in *Drosophila melanogaster*. The FEBS Journal.

[bib36] Johnson RA, Rutowski RL (2022). Color, activity period, and eye structure in four lineages of ants: Pale, nocturnal species have evolved larger eyes and larger facets than their dark, diurnal congeners. PLOS ONE.

[bib37] Kallal RJ, LaPolla JS (2012). Monograph of nylanderia (hymenoptera: formicidae) of the world, part ii: nylanderia in the nearctic. Zootaxa.

[bib38] Khila A, Abouheif E (2010). Evaluating the role of reproductive constraints in ant social evolution. Philosophical Transactions of the Royal Society B.

[bib39] Koch S, Abouheif E, Bishop CD, Hall BK (2019). Deferring Development.

[bib40] Kohout RJ (2006). Review of polyrhachis (cyrtomyrma) forel (hymenoptera: formicidae: formicinae) of Australia, borneo, new guinea and the solomon Islands with descriptions of new species. Memoirs-Queensland Museum.

[bib41] Korb J (2025). Changes of division of labour along the eusociality spectrum in termites, with comparisons to multicellularity. Philosophical Transactions of the Royal Society B.

[bib42] Krapp HG (2009). Ocelli. Current Biology.

[bib43] Kumar S, Stecher G, Suleski M, Sanderford M, Sharma S, Tamura K (2024). MEGA12: molecular evolutionary genetic analysis version 12 for adaptive and green computing. Molecular Biology and Evolution.

[bib44] Laciny A, Nemeschkal HL, Zettel H, Metscher B, Druzhinina IS (2019). Caste-specific morphological modularity in the ant tribe camponotini (hymenoptera, formicidae). BMC Zoology.

[bib45] Lapolla JS (2009). Taxonomic revision of the southeast asian ant genus euprenolepis. Zootaxa.

[bib46] Lapolla JS, Brady SG, Shattuck SO (2011). Monograph of Nylanderia (Hymenoptera: Formicidae) of the World: An introduction to the systematics and biology of the genus. Zootaxa.

[bib47] LaPolla J, Hawkes P, N. Fisher J (2013). Taxonomic review of the ant genus Paratrechina, with a description of a new species from Africa. Journal of Hymenoptera Research.

[bib48] LaPolla JS, Fisher BL (2014). Then there were five: a reexamination of the ant genus Paratrechina (Hymenoptera, Formicidae). ZooKeys.

[bib49] Leal F, Cohn MJ (2018). Developmental, genetic, and genomic insights into the evolutionary loss of limbs in snakes. Genesis.

[bib50] Lenuzzi M, Witte H, Riebesell M, Rödelsperger C, Hong RL, Sommer RJ (2023). Influence of environmental temperature on mouth-form plasticity in Pristionchus pacificus acts through daf-11-dependent cGMP signaling. Journal of Experimental Zoology. Part B, Molecular and Developmental Evolution.

[bib51] Li R, Dai X, Zheng J, Larsen RS, Qi Y, Zhang X, Vizueta J, Boomsma JJ, Liu W, Zhang G (2024). Juvenile hormone as a key regulator for asymmetric caste differentiation in ants. PNAS.

[bib52] Lynch JA, Brent AE, Leaf DS, Pultz MA, Desplan C (2006). Localized maternal orthodenticle patterns anterior and posterior in the long germ wasp Nasonia. Nature.

[bib53] Lynch VJ (2023). Is there a loophole in Dollo’s law? A DevoEvo perspective on irreversibility (of felid dentition). Journal of Experimental Zoology. Part B, Molecular and Developmental Evolution.

[bib54] MacKay WP, Mackay E (2002). The Ants of New Mexico (Hymenoptera: Formicidae).

[bib55] MacMillan O, Singer J, Perrakis S, Craig A, Ntanga D, Qiu D, Rajakumar R (2025). Juvenile hormone signalling underlies the switchpoint and differentiation of soldiers in *Camponotus Floridanus*. bioRxiv.

[bib56] Mann WM (1920). Ant guests from fiji and the british solomon islands. Annals of the Entomological Society of America.

[bib57] McAreavey JJ (1957). Revision of the genus stigmacros forel. Memoirs of the National Museum of Victoria.

[bib58] Mishra AK, Fritsch C, Voutev R, Mann RS, Sprecher SG (2021). Homothorax controls a binary Rhodopsin switch in *Drosophila ocelli*. PLOS Genetics.

[bib59] Miura T (2005). Developmental regulation of caste‐specific characters in social‐insect polyphenism. Evolution & Development.

[bib60] Monnin T, Peeters C (2008). How many gamergates is an ant queen worth?. Die Naturwissenschaften.

[bib61] Moser JC, Reeve JD, Bento JMS, Della Lucia TMC, Cameron RS, Heck NM (2004). Eye size and behaviour of day‐ and night‐flying leafcutting ant alates. Journal of Zoology.

[bib62] Narendra A, Ramirez-Esquivel F, Ribi WA (2016). Compound eye and ocellar structure for walking and flying modes of locomotion in the Australian ant, Camponotus consobrinus. Scientific Reports.

[bib63] Narendra A, Ribi WA (2017). Ocellar structure is driven by the mode of locomotion and activity time in *Myrmecia* ants. The Journal of Experimental Biology.

[bib64] Nijhout HF (2003). Development and evolution of adaptive polyphenisms. Evolution & Development.

[bib65] Passera L (1976). Origine des intercastes dans les sociétés dePheidole pallidula (Nyl.) (Hymenoptera Formicidae) parasitées parMermis sp. (Nematoda Mermithidae). Insectes Sociaux.

[bib66] Peters RS, Krogmann L, Mayer C, Donath A, Gunkel S, Meusemann K, Kozlov A, Podsiadlowski L, Petersen M, Lanfear R, Diez PA, Heraty J, Kjer KM, Klopfstein S, Meier R, Polidori C, Schmitt T, Liu S, Zhou X, Wappler T, Rust J, Misof B, Niehuis O (2017). Evolutionary history of the hymenoptera. Current Biology.

[bib67] Powell S, Price SL, Kronauer DJC (2020). Trait evolution is reversible, repeatable, and decoupled in the soldier caste of turtle ants. PNAS.

[bib68] Qiu B, Dai X, Li P, Larsen RS, Li R, Price AL, Ding G, Texada MJ, Zhang X, Zuo D, Gao Q, Jiang W, Wen T, Pontieri L, Guo C, Rewitz K, Li Q, Liu W, Boomsma JJ, Zhang G (2022). Canalized gene expression during development mediates caste differentiation in ants. Nature Ecology & Evolution.

[bib69] Ragsdale EJ, Müller MR, Rödelsperger C, Sommer RJ (2013). A developmental switch coupled to the evolution of plasticity acts through a sulfatase. Cell.

[bib70] Rajakumar R, San Mauro D, Dijkstra MB, Huang MH, Wheeler DE, Hiou-Tim F, Khila A, Cournoyea M, Abouheif E (2012). Ancestral developmental potential facilitates parallel evolution in ants. Science.

[bib71] Rajakumar R, Koch S, Couture M, Favé MJ, Lillico-Ouachour A, Chen T, De Blasis G, Rajakumar A, Ouellette D, Abouheif E (2018). Social regulation of a rudimentary organ generates complex worker-caste systems in ants. Nature.

[bib72] Rajakumar A, Pontieri L, Li R, Larsen RS, Vásquez-Correa A, Frandsen JKL, Rafiqi AM, Zhang G, Abouheif E (2024). From egg to adult: a developmental table of the ant monomorium pharaonis. Journal of Experimental Zoology. Part B, Molecular and Developmental Evolution.

[bib73] Revell LJ (2024). phytools 2.0: an updated R ecosystem for phylogenetic comparative methods (and other things). PeerJ.

[bib74] Sabat D, Priyadarsini S, Mishra M (2017). Understanding the structural and developmental aspect of simple eye of *Drosophila*: the ocelli. Journal of Cell Signaling.

[bib75] Santschi F (1936). Liste et descriptions de fourmis du Maroc. Bulletin de La Société Des Sciences Naturelles Du Maroc.

[bib76] Sayers EW, Bolton EE, Brister JR, Canese K, Chan J, Comeau DC, Connor R, Funk K, Kelly C, Kim S, Madej T, Marchler-Bauer A, Lanczycki C, Lathrop S, Lu Z, Thibaud-Nissen F, Murphy T, Phan L, Skripchenko Y, Tse T, Wang J, Williams R, Trawick BW, Pruitt KD, Sherry ST (2022). Database resources of the national center for biotechnology information. Nucleic Acids Research.

[bib77] Schindelin J, Arganda-Carreras I, Frise E, Kaynig V, Longair M, Pietzsch T, Preibisch S, Rueden C, Saalfeld S, Schmid B, Tinevez JY, White DJ, Hartenstein V, Eliceiri K, Tomancak P, Cardona A (2012). Fiji: an open-source platform for biological-image analysis. Nature Methods.

[bib78] Schultner E, Wallner T, Dofka B, Brülhart J, Heinze J, Freitak D, Pokorny T, Oettler J (2023). Queens control caste allocation in the ant *Cardiocondyla obscurior*. Proceedings. Biological Sciences.

[bib79] Schwarz S, Wystrach A, Cheng K (2011). A new navigational mechanism mediated by ant ocelli. Biology Letters.

[bib80] Schwarzkopf M, Liu MC, Schulte SJ, Ives R, Husain N, Choi HMT, Pierce NA (2021). Hybridization chain reaction enables a unified approach to multiplexed, quantitative, high-resolution immunohistochemistry and in situ hybridization. Development.

[bib81] Sharaf MR, Aldawood AS, Mohamed AA, Hita Garcia F (2020). The genus Lepisiota Santschi, 1926 of the Arabian Peninsula with the description of a new species. Journal of Hymenoptera Research.

[bib82] Shattuck S, O’Reilly A (2013). Revision of the australian endemic ant genera pseudonotoncus and teratomyrmex (hymenoptera: formicidae: formicinae). Zootaxa.

[bib83] Smith F (1858). Catalogue of Hymenopterous Insects in the Collection of the British Museum: 6: Formicidae.

[bib84] Smith MR (1947). A study of polyergus in the United States, based on the workers (hymenoptera: formicidae). American Midland Naturalist.

[bib85] Snelling RR (1979). Aphomomyrmex and a related new genus of arboreal African ants (Hymenoptera: Formicidae). Contributions in Science.

[bib86] Sommer RJ (2020). Phenotypic plasticity: from theory and genetics to current and future challenges. Genetics.

[bib87] Susoy V, Ragsdale EJ, Kanzaki N, Sommer RJ (2015). Rapid diversification associated with a macroevolutionary pulse of developmental plasticity. eLife.

[bib88] The Honeybee Genome Sequencing Consortium (2006). Insights into social insects from the genome of the honeybee *Apis mellifera*. Nature.

[bib89] Visser B, Le Lann C, den Blanken FJ, Harvey JA, van Alphen JJM, Ellers J (2010). Loss of lipid synthesis as an evolutionary consequence of a parasitic lifestyle. PNAS.

[bib90] Visser B, Alborn HT, Rondeaux S, Haillot M, Hance T, Rebar D, Riederer JM, Tiso S, van Eldijk TJB, Weissing FJ, Nieberding CM (2021). Phenotypic plasticity explains apparent reverse evolution of fat synthesis in parasitic wasps. Scientific Reports.

[bib91] Vizueta J, Xiong Z, Ding G, Larsen RS, Ran H, Gao Q, Stiller J, Dai W, Jiang W, Zhao J, Guo C, Zhang X, Zuo D, Zhong W, Schiøtt M, Liu C, Zhang H, Dai X, Andreu I, Shi Y, Tretter S, He D, Gautam S, Li Z, Hickey G, Ivens ABF, Meurville MP, Hita-Garcia F, Kass JM, Guénard B, Moreau C, Paten B, LeBoeuf AC, Economo EP, Chapuisat M, Shik JZ, Ward PS, Heinze J, Schultz TR, Li Q, Dunn RR, Sanders NJ, Liu W, Schrader L, Boomsma JJ, Zhang G, GAGA Consortium (2025). Adaptive radiation and social evolution of the ants. Cell.

[bib92] Vong E, Parisien S, Orfali H, Rajakumar R (2025). Evolution and regulation of decoupled dimorphic scaling patterns in ants. bioRxiv.

[bib93] Ward PS (2001). Taxonomy, phylogeny and biogeography of the ant genus *Tetraponera* (Hymenoptera: Formicidae) in the Oriental and Australian regions. Invertebrate Taxonomy.

[bib94] Ward PS (2005). A synoptic review of the ants of California (Hymenoptera: Formicidae). Zootaxa.

[bib95] Ward PS (2014). The phylogeny and evolution of ants. Annual Review of Ecology, Evolution, and Systematics.

[bib96] Werner MS, Claaßen MH, Renahan T, Dardiry M, Sommer RJ (2018). Adult influence on juvenile phenotypes by stage-specific pheromone production. iScience.

[bib97] West-Eberhard MJ (1989). Phenotypic plasticity and the origins of diversity. Annual Review of Ecology and Systematics.

[bib98] West-Eberhard MJ (2003). Developmental Plasticity and Evolution.

[bib99] Wheeler W (1917). The Australian ant-genus Myrmecorhynchus (Ern. André) and its position in the subfamily Camponotinae. Transactions of the Royal Society of South Australia.

[bib100] Williams JL, Lapolla JS (2016). Taxonomic revision and phylogeny of the ant genus *Prenolepis* (Hymenoptera: Formicidae). Zootaxa.

[bib101] Yorimitsu T, Kiritooshi N, Nakagoshi H (2011). Defective proventriculus specifies the ocellar region in the *Drosophila* head. Developmental Biology.

[bib102] Zattara EE, Macagno ALM, Busey HA, Moczek AP (2017). Development of functional ectopic compound eyes in scarabaeid beetles by knockdown of *orthodenticle*. PNAS.

[bib103] Zimmerman JE, Bui QT, Liu H, Bonini NM (2000). Molecular genetic analysis of *Drosophila* eyes absent mutants reveals an eye enhancer element. Genetics.

