# Supplementary material for: Latent gene network expression underlies partial re-evolution of a polyphenic trait in the worker caste of ants
Source: eLife. 2026 Aug 18;15:RP110148. doi: 10.7554/eLife.110148 (PMC13485301; doi:10.7554/eLife.110148)
Supplement: Peer review history

# Editor report: eLife Assessment

**DOI:** 10.7554/eLife.110148.3.sa0

**Authors:** Vincent J Lynch

**Affiliations:** University at Buffalo, State University of New York United States

**Keywords:** Convincing, Important

This **important** study explores whether complex structures that are lost during evolution can re-evolve, which is a long-standing debate in evolutionary and developmental biology. The authors demonstrate that re-evolution can occur if the gene regulatory network that underlies the development of complex traits is maintained. The evidence supporting its conclusions is **convincing** and the work will be of interest to those studying the evolution and development of complex traits.

# Referee report: Reviewer #2 (Public review)

**DOI:** 10.7554/eLife.110148.3.sa1

Summary:

The manuscript titled "Latent gene network expression underlies partial re-evolution of a polyphenic trait in the worker caste of ants" by Vasquez-Correa et al. aimed to study genetic mechanisms underlying developmental plasticity, especially binary polyphenism in queen vs worker ant castes. This is an interesting question regarding the extent to which phenotypic traits were altered, lost or regained, and how molecular pathways (upstream vs. downstream) can facilitate this process.

In ants, reproductive castes (queens and males) develop wings as well as 3 ocelli for mating flights and other activities, while worker castes are wingless, and in some species, they have either no or a reduced number of ocelli. The phylogenetic analysis showed that in the Camponotini ant clade, the one-ocellus phenotype re-evolved in three species independently. The authors analyzed the conserved developmental pathways between *Drosophila* (well-established) and ants using HCR (a high-quality in situ hybridization technique). They found that although upstream genes for the development of ocelli (otd and hh) showed similar expression between castes, downstream genes (toy, eya, and so) had reduced or no expression in workers of C. floridanus, and this differential expression may lead to partial or complete loss of ocelli. Consistently, workers develop rudimentary tissues, suggesting that they initiate the ocellus developmental process but somehow stop it before adulthood.

Strengths:

Evo-devo approaches to reveal conserved molecular pathways of ocellus development. High-quality HCR provided convincing evidence of the expression of key genes in ocelli, eyes and antenna throughout larval development.

Using HCR, the authors showed differential expression of downstream genes in males vs. soldiers vs. minor workers of C. floridanus, which might explain phenotypic differences between castes.

Comments on revised version.

The authors have addressed the concerns in the revision. No further comments.

# Referee report: Reviewer #3 (Public review)

**DOI:** 10.7554/eLife.110148.3.sa2

Summary:

This paper examines the loss and re-evolution of specific organs during the evolution of ants. The authors show that these organs, the ocelli, disappear and are re-evolved in different ant species, and in different ant castes within these species. The Authors show that this is linked to a conserved GRN discovered in *Drosophila*, that appears to underlie the development of the ocelli, and demonstrate that this GRN appears to remain active in the developing heads of ants that have no ocelli- implying that it is the evolutionary latency of this GRN that allows loss and subsequent evolution.

Strengths:

This manuscript has outstanding imaging of a very difficult developing organ, and the key data, fluorescence in situ hybridisation, is done well and clearly shows what the authors wish to demonstrate. The methods are well described and underpin the whole work.

The authors convincing demonstrate that gene expression patterns imply the conservation of the ocellus gene regulatory network from *Drosophila* to ants. They further show that this network is present even in ants that don't produce an adult ocellus, but do show that in those species, loss of a developing nascent ocellus (which they identify) occurs at the same time as an interruption in the expression of the key genes in the GRN. All of this data is beautifully presented and explained.

Weaknesses:

There is one key weakness in that there are no functional students that indicate that the GRN actually does make the ocellus, though the expression patterns are convincing. This applies to loss of the ocellus as well. It would be nice to see that transient loss of the ocelli GRN might lead to loss of ocelli in ant species that have them. These are very difficult things to achieve as the key genes have earlier developmental roles, such that CRISPr knockouts would not be interpretable, and transient RNAi in the head capsules of developing pupal ants would be challenging.

As the authors note in their response this is very difficult to achieve. While the addition of this data would raise this manuscript to an outstanding one, I think the data presented is solid, well-presented and provides novel insight.

# Author comment: Author response

**DOI:** 10.7554/eLife.110148.3.sa3

**Authors:** Angelly Vasquez-Correa, Johanna Arnet, Travis Chen, Ehab Abouheif

**Affiliations:** Women's Hospital, School of Medicine, Zhejiang University Hangzhou China; McGill University Montreal Canada; Women's Hospital, School of Medicine, Zhejiang University Hangzhou China; Women's Hospital, School of Medicine, Zhejiang University Hangzhou China

The following is the authors’ response to the original reviews.

**Public Reviews:**

**Reviewer #1 (Public review):**

Summary:

The manuscript by Vasquez-Correa and colleagues describes the expression pattern of the ocelli (simple eye) gene regulatory network in ants. They correlate the expression pattern of these genes with the presence and absence of ocelli in different classes and species of ants. The presence of ocelli is a polyphenic trait in ants - understanding the molecular and developmental underpinnings of polyphenic traits is of significant interest to evolutionary biologists, developmental biologists, and ecologists. The authors propose that the presence of the latent expression of the ocellar network in classes of ants that do not display ocelli in the adults may underlie the re-evolution of ocelli within the ant lineage.

Strengths:

The strengths of the manuscript are that it is well written, the images are of the highest quality, and the data support the conclusions of the authors.

We thank Reviewer 1 for their positive comments.

Weaknesses:

One improvement that could be made is to include imaginal discs of the queen ants as well as scanning electron images of the ocelli of the queen ant to match the pupal stage images of the worker and soldier ants. A second improvement is to attempt a gene knockdown using RNAi or similar methods to ensure that the genes that are being studied are, in fact, responsible for ocelli development in the ant.

The reproductive caste in ants is typically composed of both winged males and winged queens. We agree with Reviewer 1 that the queen caste, which develop 3 fully functional ocelli, is an important point of comparison in our study to the wingless minor workers and soldiers. Unfortunately, however, laboratory colonies rarely produce reproductive queens, and in the field, queen production in colonies of *C. floridanus* occurs within a narrow seasonal window, making the collection of queen larvae particularly challenging for developmental work. In contrast, the winged males, which also develop 3 functional ocelli like the queens for help during mating flights, can be readily generated in the lab throughout the year. Therefore, we use males as a proxy for characterizing ocelli development and GRN in queens and the winged reproductive caste as a whole. Given the deeply conserved gene regulatory networks underlying this trait across insects, we believe this is a reasonable assumption.

We also agree with Reviewer 1 that using RNAi to knock down genes in the ocelli GRN would improve the study. For completeness of the scientific record, we would like reviewers and readers to know that we actually did, in fact, invest significant effort trying to knock down *otd-1* (ortholog of the *Drosophila otd* gene), which functions as key upstream regulator of ocellar development. In *Drosophila*, RNAi knockdown of *otd* disrupts the development of all three ocelli as well as fine morphological features on the anterior of the head. In *C. floridanus*, *otd -1* is expressed in the head capsule and brain (see Author response image 1 in this response). Injection of dsRNA of *otd*-1into whole soldier-destined larvae, significantly reduced *otd -1* expression in the brain relative to its control, while in the head capsule, *otd -1* expression remained largely unchanged relative to its control (see Author response image 1 in this response). This indicates that in the same individual, the injected *otd -1* dsRNA was able to penetrate and significantly reduce *otd -1* expression in the brain, but, was unable to penetrate the head capsule, where *otd -1* expression remained largely unchanged. No ocellar phenotypes could be observed in pupae or adults. Therefore, for technical (not biological) reasons, we were unable to knockdown genes in the ocelli GRN in the head capsule. We hope to solve this technical problem in the coming years to add a mechanistic explanation for the latent expression and maintenance of the ocelli GRN in workers that completely lack ocelli as adults.

**Reviewer #2 (Public review):**

Summary:

The manuscript titled "Latent gene network expression underlies partial re-evolution of a polyphenic trait in the worker caste of ants" by Vasquez-Correa et al. aimed to study genetic mechanisms underlying developmental plasticity, especially binary polyphenism in queen vs worker ant castes. This is an interesting question regarding the extent to which phenotypic traits were altered, lost or regained, and how molecular pathways (upstream vs. downstream) can facilitate this process.

In ants, reproductive castes (queens and males) develop wings as well as 3 ocelli for mating flights and other activities, while worker castes are wingless, and in some species, they have either no or a reduced number of ocelli. The phylogenetic analysis showed that in the Camponotini ant clade, the one-ocellus phenotype revolved in three species independently. The authors analyzed the conserved developmental pathways between *Drosophila* (well-established) and ants using HCR (a high-quality in situ hybridization technique). They found that although upstream genes for the development of ocelli (otd and hh) showed similar expression between castes, downstream genes (toy, eya, and so) had reduced or no expression in workers of C. floridanus, and this differential expression may lead to partial or complete loss of ocelli. Consistently, workers develop rudimentary tissues, suggesting that they initiate the ocellus developmental process but somehow stop it before adulthood.

Strengths:

Evo-devo approaches to reveal conserved molecular pathways of ocellus development. High-quality HCR provided convincing evidence of the expression of key genes in ocelli, eyes and antenna throughout larval development.

Using HCR, the authors showed differential expression of downstream genes in males vs. soldiers vs. minor workers of C. floridanus, which might explain phenotypic differences between castes.

We thank Reviewer 2 for their positive comments.

Weaknesses:

Although the molecular pathway is conserved, the mechanism underlying the lack of ocelli in workers remains unclear. In C. floridanus, it could be explained by the evidence of no expression of certain developmental genes, but in other species, e.g. Polyrachis rastellata, is their expression intact, or reduced? There is no control male.

In addition, HCR in species with partial re-evolution (if their genomes have been sequenced) would be useful to understand the mechanism. For example, there might be differential spatial expression between medial and lateral ocelli.

We agree with Reviewer 3 that investigating the mechanisms underlying the lack of specific ocelli in these and other species is the next step for this research. Here, our main focus was instead on trying to explain the mechanisms underlying partial reversion of ocelli through the persistence of ocelli GRN expression in adult workers lacking ocelli. We therefore focused on the latent expression of the ocelli GRN in *Polyrachis rastellata*, a species that completely lack ocelli in adult workers, and how it may have facilitated the partial reversion of a single ocellus in its congener *Polyrachis bihamata*. Therefore, although we did not reveal specific interruption points in the ocelli GRN in *Polyrachis rastellata*, our results showing that this species expresses three genes of the ocelli GRN, offers sufficient evidence that this network is conserved and likely facilitated the partial reversion to a single ocellus in *P. bihamata*.

We also agree with Reviewer 3 regarding the male control in *P. rastellata* and obtaining the species in our study that have undergone partial re-evolution. Unfortunately, these ants occur in Southeast Asia and are very difficult to collect. For males in *P. rastellata*, our colony died before we could try to induce male development. However, given the deep conservation of the network in the males of a genus within the same subfamily (Camponotini), we feel it is reasonable to assume that the network would also be conserved in the males of *P. rastellata*, especially since the genes we sampled are conserved in workers that do not develop ocelli as adults. As am sure the Reviewer may know that this is a continual challenge of working with emerging models in evodevo.

**Reviewer #3 (Public review):**

Summary:

This paper examines the loss and re-evolution of specific organs during the evolution of ants. The authors show that these organs, the ocelli, disappear and are re-evolved in different ant species and in different ant castes within these species. The authors show that this is linked to to a conserved GRN discovered in *Drosophila*, that appears to underlie the development of the ocelli, and demonstrate that this GRN appears to remain active in the developing heads of ants that have no ocelli- implying that it is the evolutionary latency of this GRN that allows loss and subsequent evolution.

Strengths:

This manuscript has outstanding imaging of a very difficult developing organ, and the key data, fluorescence in situ hybridisation, is done well and clearly shows what the authors wish to demonstrate. The methods are well described and underpin the whole work.

The authors convincing demonstatrate that gene expression patterns imply the conservation of the ocellus gene regulatory network from *Drosophila* to ants. They further show that this network is present even in ants that don't produce an adult ocellus, but do show that in those species, loss of a developing nascent ocellus (which they identify) occurs at the same time as an interruption in the expression of the key genes in the GRN. All of this data is beautifully presented and explained.

We thank Reviewer 3 for their positive comments.

Weaknesses:

There is one key weakness in that there are no functional students that indicate that the GRN actually does make the ocellus, though the expression patterns are convincing. This applies to loss of the ocellus as well. It would be nice to see that transient loss of the ocelli GRN might lead to loss of ocelli in ant species that have them. These are very difficult things to achieve, as the key genes have earlier developmental roles, such that CRISPR knockouts would not be interpretable, and transient RNAi in the head capsules of developing pupal ants would be challenging.

We agree with Reviewer 3 that functional experiments in species where workers both have ocelli present and absent is a key next step in this research. Please see our response to Reviewer 1 on our failed attempts to achieve this. We are therefore grateful to Reviewer 3 for acknowledging the challenges in trying to establish RNAi and CRISPR in the head capsules of developing workers in these ants. Also, please see our response to Reviewer 2 on the difficulty of finding and collecting these ants, which occur mainly in Southeast Asia.

**Recommendations for the authors:**

**Reviewer #1 (Recommendations for the authors):**

One improvement that could be made is to include imaginal discs of the queen ants as well as scanning electron images of the ocelli of the queen ant to match the pupal stage images of the worker and soldier ants.

A second improvement is to attempt a gene knockdown using RNAi or similar methods to ensure that the genes that are being studied are in fact responsible for ocelli development in the ant.

Please see our response to Reviewer 1 above.

**Reviewer #2 (Recommendations for the authors):**

For the questions below, if there is no experimental evidence, consider addressing them in the Discussion.

Do sizes of ocelli different between castes? For example, even workers have 1-3 ocelli, their sizes are smaller than those of males/queens, especially in workers with 1 ocellus. If so, might it be continuous (not binary) changes in downstream gene expression that control ocellus size, with no ocellus below threshold? Does this favor the hypothesis of threshold but not switch?

We thank Reviewer 3 for highlighting an important point about the size and development of ocelli. Observations suggest that ocelli tend to be larger in queens and males than in workers and soldiers in species with ocelli. However, we lack quantitative data to test this conclusively. We now include a sentence on the Discussion stating that an important avenue of future work should investigate whether threshold or switch mechanisms influencing the presence/absence, as well as size, of ocelli between queens and workers.

For the species whose workers have a single ocellus, are there variations, e.g. spanning from 0, 1 to 2? If 2, always one medial plus one of the two laterals? If always one, it would be a good control for staining to see up- vs down-regulation of downstream gene expression within the same individual.

We agree with Reviewer 2 that this is a fascinating approach to our question. We have not observed natural wild-type variation in the number of developing ocelli in the same-sized individuals in the worker caste. However, in a distantly related leaf-cutting ant species (*Atta cephalotes*) belonging to different subfamily (the Myrmicinae) individuals with different head-to-body scaling within the same colony can vary in the number of ocelli. For example, soldiers of *Atta cephalotes* include individuals developing one, two, or three ocelli. These configurations can appear as only the median ocellus, only the two lateral ocelli, or even the median plus a single lateral ocellus. Interestingly, these correlations vary with changes in the size and head-to body scaling, suggesting that each ocellus can undergo different degrees of development, with one or more remaining vestigial or completely absent. On the other hand, workers in other species consistently develop a single ocellus, like in workers of *Polyrachis bihamata*, with no correlation to size or head-to-body scaling. These cases highlight how evolutionarily labile this trait is among workers of different ant species, which supports our proposal that the underlying gene regulatory network remains latent, thereby facilitating the emergence of novel trait combinations. We therefore agree on the importance of comparing the developmental mechanisms underlying these patterns temporally across larval stages and between individuals within a colony. We have now incorporated 2 sentences into the discussion, stating that this will be an important avenue for future work.

Is there any function of a single ocellus in workers, or just a consequence of incomplete down-regulation of gene expression?

Thank you again for highlighting these important points that help us to elaborate on the discussion of our study. The functional role of ocelli in species that develop these structures remains largely understudied. However, for some species particularly within the Formicinae clade the function of the three ocelli in workers has been investigated, revealing that they serve as a celestial compass that facilitates navigation. We reference these findings in our Introduction and Discussion to illustrate that the presence of three ocelli in workers can represent an adaptive trait. In contrast, the functional significance of a single ocellus or of partially developed ocelli remains an important question. This knowledge gap presents a promising avenue for future research to understand the adaptive value of reduced, partially suppressed ocellar development. We have now added a sentence in the discussion stating this.

In previous studies, JH treatment can increase the number of ocelli in workers, consistent with its role in promoting reproductive development. In the ocellus developmental pathway, what causes the reduction of downstream gene expression in C. floridanus? Does JH directly regulate their expression?

We thank Reviewer 2 for proposing yet another interesting question for future investigation, which we have added to the Discussion.

The only current evidence available in *C. floridanus* is a recent study (MacMillan et al. 2025), in which minor workers and soldiers were treated with JH at different developmental stages. Unfortunately, no evidence of ocelli induction was observed in JH-treated individuals, suggesting that the mechanisms of ocelli development in *C. floridanus* might be highly canalized, especially in species that exhibit worker polymorphism (inter-individual variation in size and head-to-body scaling within the worker cate). However, more studies are required to understand why in *Monomorium pharonis* (no worker polymorphism) ocelli development can be readily induced by JH, while in another *C. floridanus* (with worker polymorphism) it appears quite difficult.

"In *D. melanogaster*, the head develops from the eye-antenna disc" This statement is not correct. The brain does not belong to the eye-antennal disc.

We thank Reviewer 2 for catching the misspelling. We have changed the name to eye-antenna disc in the sentence.

**Reviewer #3 (Recommendations for the authors):**

It is hard to see the developing ocelli in Figure 7 - could the authors increase the contrast to make them more visible?

We have made the suggested changes to Figure 7 in the main article, and it has indeed improved the figure.

**Author response image 1.** RNAi knockdowns in developing soldiers of *Camponotus floridanus* show a reduction of *otd -1* expression in the brain, but no effect on *otd -1* expression in the eye-antenna disc. A. HCR revealing *otd -1* expression in the brain B. qPCR of *otd -1* expression after RNAi knockdown shows significantly reduced *otd -1* expression in the brain, C. HCR revealing *otd -1* expression in the eye-antenna disc D. qPCR of *otd -1* expression after RNAi knockdown shows no significant affect on *otd -1* expression in the eye-antenna disc.
